# Female Corporality, Gender Roles, and Their Influence on Women's Mental Health in Times of COVID-19

**DOI:** 10.3389/fgwh.2020.563209

**Published:** 2020-11-12

**Authors:** Margarita Sáenz-Herrero, Mayte López-Atanes, María Recio-Barbero

**Affiliations:** ^1^Department of Psychiatry, Cruces University Hospital, Barakaldo, Spain; ^2^Department of Psychiatry, Biocruces Bizkaia Health Research Institute, Barakaldo, Spain

**Keywords:** corporality, gender, mental health, pandemic, COVID-19

## Introduction

The German language distinguishes two distinct words for “the body.” *Körper*, which posits the body as an object, and *Leib*, or the experienced reality of embodiment (1). These two forms of acknowledging the body are not as well-expressed in Spanish or English. Ortega y Gasset called the former “extra-body” and the latter “intra-body” (2). The “intra-body” relates to the phenomenological concept of corporality, which refers to the sense of embodiment, and does not represent something anatomical as does the body. Corporality has been studied in philosophy and other disciplines such as psychiatry, feminist psychology (3), and neurosciences (4).

Western society is grounded on a dichotomous view that can be dated back to Plato's metaphysics, closely related to Descartes thinking, where an immaterial spirit is separated from a material substance, which is the body (5, 6). The spirit, reason, and Cartesian logos go invariably behind the subtle and perishable body. As we live in a male-centered and male-identified patriarchal society, the masculine concept is intimately related to reason, to the Cartesian logos, while the body is identified with the feminine (7). From an anthropological perspective, Nature is represented as feminine and subordinated to Culture, which is mainly masculine. Likewise, Reason and Mind are related to masculinity, while Body and Nature are feminine (8).

Patriarchy is based on this anthropological meaning of the body as something identified with femineity (7). Consequently, in Western societies being a man or a woman means a different role of the body in the construction of the own identity, and this creates different interactions with the others (6). Women are acculturated to build their self-image using the eyes of others as the primary view of the physical selves (3, 9), to the point that some authors such as Susie Orbach argue this fact makes women ending up seeing their body as commodities within a consumerist culture (9). Besides anthropological and philosophical theories, psychological research has proven that women, compared to men, focus more on their own bodies' aesthetic features and not on the functional ones (10). Even in childhood, girls are already more conscious about their body weight and appearance than their male counterparts (11).

Several factors have been discussed as potential causes for these differences, in particular gender roles. Femineity contains traits and behaviors related to caregiving, love, and a stereotypical thin body ideal as core factors, all of which constitute a part of the feminine gender role. We live in a gendered society in which there are polarized expectations of the behavior of females and males (12). These gender-stereotyped body image ideals lead to unjustified importance of the body in the social well-being of women, as well as self-objectification and an increased risk of eating disorders to get the thin ideal (13).

## Influence of the Pandemic on Corporality

The current pandemic scenario has challenged our own bodies' perception since part of society became aware of their body through the disease process. By experiencing pain, fever, discomfort, and distress, people comprehend how their bodies influence their well-being and identity. In fact, illness can affect self-stem, self-perception, and change body image (14). As women's identity may be more susceptible to being influenced by their physical appearance, the coronavirus pandemic will probably distress women to a greater extent by focusing even more on their vulnerabilities and increasing their body awareness. It has been previously reported that a self-critical attitude against the body and body dissatisfaction are predictors for developing eating disorders (15). Therefore, in these circumstances, an increase in women's incidence of eating disorders could also be expected.

The connection between the body and mental health is bidirectional. Mental distress can interfere with how people perceive their physical sensations, and on the other hand, illness and body dissatisfaction can act as triggers for mental pathology (14, 16–19). The link between body awareness and psychopathology culminates in mental disorders like anxiety and somatic symptom disorders (SSD), in which the cognitive appraisal of somatic symptoms is distorted, and patients catastrophize normal physiological sensations (20). This attitude leads to more anxiety. The COVID-19 pandemic, through increased body awareness, is likely to worsen both anxiety disorders and SSD, as these patients are already distorting their somatic sensations and misinterpreting them as dangerous. As these pathologies have been described to be more prevalent in women, reaching an estimated female-to-male ratio of up to 10:1 in SSD (21), we can presume that women will be affected to a greater extent.

## Influence of the Pandemic on Gender Roles

Ongoing worldwide pandemic has not only made women more aware of their bodies but also their gender roles. In our culture, female subjectivity is constructed concerning the body, caregiving, and love for the others. Other values, such as sacrifice, effort, affection, and suffering, which are all also associated with the female gender role, are a breeding ground for psychological distress, especially in times of a pandemic. Following the theoretical picture of gender roles, available data show that women contribute to 71% of the global hours of informal care (22), a task that became essential during the mandatory confinement imposed in most countries. As a result, work-life balance has been dramatically affected by closing schools and childcare centers, which significantly burdens working mothers. We can therefore infer that somehow the pandemic has accentuated gender roles by imposing greater responsibility on women. As previous research has shown, gender roles and informal cares are sources of distress and psychosocial exhaust for women (23, 24). An increase in these duties will undoubtedly have long-term implications for mental health that have not yet been objectified by ongoing studies.

## Conclusions

The corporality of women is closely influenced by the female gender role. Gender roles are cultural constructs developed within a male-identified patriarchal culture that identifies femineity with the cult of the body. During the coronavirus pandemic, our androcentric society became aware of its futility, of the human body's fragility through the experience of illness. Women, which were already at a higher risk of developing mental health issues, and increased body awareness may produce more significant psychological distress than men. Parallelly, the accentuation of gender roles by an increased need for caregiving during the confinement can also impact women's mental health, as they are the leading providers of informal care.

Although more research is needed to establish the psychological impact on women, there is enough data to hypothesize that the pandemic will distress women to a greater extent. Therefore, gender-sensitive interventions during the pandemic should be considered, along with psychological interventions that address body awareness. Given this situation, public policies should promote equity in care and strengthen those research programs that include a gender perspective. This is the moment to invest in women's mental health.

## Author Contributions

All authors listed have made a substantial, direct and intellectual contribution to the work, and approved it for publication.

## Conflict of Interest

The authors declare that the research was conducted in the absence of any commercial or financial relationships that could be construed as a potential conflict of interest.
